# Core blood biomarkers of Alzheimer's disease: A single-center real-world performance study

**DOI:** 10.1016/j.tjpad.2024.100027

**Published:** 2025-01-01

**Authors:** Federico Emanuele Pozzi, Elisa Conti, Giulia Remoli, Niccolò dell'Orto, Simona Andreoni, Fulvio Da Re, Gessica Sala, Luca Cuffaro, Carlo Ferrarese, Ildebrando Appollonio, Chiara Paola Zoia, Lucio Tremolizzo

**Affiliations:** a Neurology, Fondazione IRCCS “San Gerardo dei Tintori”, Monza, Italy; b Milan Center for Neuroscience (NeuroMI), University of Milano-Bicocca, Monza, Italy; c Laboratory of Neurobiology, School of Medicine and Surgery, University of Milano-Bicocca, Monza, Italy

**Keywords:** Plasma biomarkers, Dementia, Alzheimer's disease, Tau, Amyloid, BBAD

## Abstract

**Background:**

The new criteria for Alzheimer's disease pave the way for the introduction of core blood biomarkers of Alzheimer's disease (BBAD) into clinical practice. However, this depends on the demonstration of sufficient accuracy and robustness of BBADs in the intended population.

**Objectives:**

To assess the diagnostic performance of core BBADs in our memory clinic, comparing them with cerebrospinal fluid (CSF) analysis.

**Design:**

Real-world cross-sectional observational study.

**Setting:**

Memory Clinic of Fondazione IRCCS “San Gerardo dei Tintori,” Monza, Italy.

**Participants:**

*n* = 102 consecutive outpatients (mean age: 71.0 ± 7.6 years) with cognitive impairment undergoing routine lumbar puncture.

**Measurements:**

CSF Aβ40, Aβ42, tTau, and pTau181 levels were measured. Plasma biomarkers were evaluated using Lumipulse® G600II. Logistic regression and Receiver Operating Characteristic (ROC) analysis were used to assess biomarker performance. The diagnosis of Alzheimer's disease was based on CSF Aβ42/40 ratio.

**Results:**

Plasma pTau217 demonstrated the highest diagnostic accuracy (AUC=0.91), followed by pTau181 (AUC=0.88) and Aβ42/40 (AUC=0.83). In robustness analyses, only pTau217 and pTau181 performance remained consistent, while that of Aβ42/40 ratio declined with added random variability. pTau217 significantly outperformed other BBAD, with the exception of pTau181. pTau BBAD were significant predictors of baseline Mini-Mental State Examination scores.

**Conclusions:**

Plasma pTau217, measured with Lumipulse®, is a robust and reliable BBAD for detecting amyloid pathology in a memory clinic setting, offering a practical and less invasive alternative to traditional CSF testing.

## 1 Introduction

Aging is the major risk factor for Alzheimer's dementia (AD). With the progressive aging of the population, we are prone to face in the next years a major increase in AD prevalence worldwide. Dementia due to Alzheimer's disease is now considered as the tip of the iceberg of a process starting many years (probably decades) before, with beta-amyloid (Aβ)/Tau proteinopathy and neuroinflammation harboring secretly under the ashes. While we may not succeed in curing Alzheimer's as a disease, the efforts to prevent the clinical maturation of AD may take advantage of such a long-time window [1]. One of the most relevant preliminary requirements consists in the real-world availability of a reliable, accessible and inexpensive way of stratifying risk. Conceivably, core markers of AD proteinopathy, such as Aβ and Tau isoforms are to be preferred, since they more closely reflect the specificity of the underlying neuropathological process. What is more, according to the current research frame, the Alzheimer's continuum is defined as a biological construct with respect to the presence of amyloid, phosphorylated tau and neurodegeneration (AT_1_-T_2_ N profile), further implying a debated shift of attention to the preclinical stages of the disease [2].

Cerebrospinal fluid (CSF) core markers of AD include Aβ, and phospho-tau (pTau) protein levels: robust evidence indicates that Aβ42/40 ratio decrease, and pTau and total Tau (tTau) increase in the CSF, reliably marking Alzheimer's continuum. While fully implemented into clinical practice, lumbar puncture remains an invasive way, albeit minimally, of getting information in AD. Blood biomarkers of Alzheimer's disease (BBAD) gained in the recent years much attention, since they potentially meet all the proposed requirements for supporting preventive strategies in the general population setting.

BBAD core markers include plasma Aβ42 and Aβ40, their ratio, and the more promising pTau181 and pTau217, among other phosphorylated isoforms of the microtubule-associated protein Tau. Although the exact source of these plasma proteins has never been completely elucidated, they have been proposed to reflect, at least in part, those central processes characterizing AD, plausibly marking the spread of neuronal damage, and correlating to the CSF A status and amyloid-PET positivity. Plasma Aβ42 and Aβ42/40 ratio, initially deemed as unreliable, have gained more attention along the years, although many confounding issues still limit their potential use in clinical settings [3]. On the other hand, plasma pTau immunoassays measure large-fold changes across the Alzheimer's continuum, including pre-symptomatic stages. One question which remains in part still open regards which pTau will perform better in the clinical setting, being currently pTau217 the one characterized by the highest discriminative power between *A*+ and A- subjects [4,5], and possibly able to predict the development of cognitive impairment [6]. pTau181 also displays reliable performances in this sense, and many others are currently investigated, such as pTau231 [3,4,7]. A combinations of BBADs may be even more accurate in detecting *A*+ subjects [8].

BBAD research is running also beyond the AD-core, and neurofilament light chain (NfL) is also an interesting candidate for an Alzheimer diagnostic panel, since it strictly results from factual axonal damage, and it is plausibly very sensitive to disease progression, albeit lacking real specificity. In the latest criteria, NfL represent a biomarker of N [2].

According to these research premises, in the future, any community-dwelling subject might theoretically (and even plausibly) undergo as an outpatient to a simple blood withdrawal, obtaining in a cheap and fast way a complete AT profile, which might be of use for implementing AD preventing strategies. The real-world value of these biomarkers still needs to be fully assessed before implementing their widespread use outside the frame of research.

The aim of the present study consisted in testing the diagnostic value of a BBAD panel measured by the widely available Lumipulse® G System in a consecutive outpatient sample accessing our Memory Clinic for cognitive decline, ranging from mild cognitive impairment to full-blown dementia, and contextually undergoing lumbar puncture for the ATN CSF profiling. Besides plasma Aβ42, Aβ40 and NfL, both pTau181 and pTau217 were included to compare their performances head-to-head.

## 2 Methods

### 2.1 Inclusion criteria

In this retrospective real-world study, we analyzed blood and CSF biomarkers of AD in consecutive outpatients undergoing routine diagnostic lumbar puncture for cognitive impairment at our Center for Dementia and Cognitive Decline at the Fondazione IRCCS “San Gerardo dei Tintori”, Monza (Italy), since December 2023. These patients were also enrolled in the Against-AD and CAPE (NCT05756270) studies and signed an informed consent; patients with significant cognitive decline were recruited either if a legal representative was available, or if they were judged able to understand the study aims by the MacArthur Competence assessment tool for clinical research [9]. Clinical charts were later reviewed to extract diagnostic information, including diagnoses coded as mild cognitive impairment (MCI) or dementia, as well as data on age, sex, and Mini-Mental State Examination (MMSE) scores. MCI diagnoses were made according to the 2011 NIA-AA criteria [10], while dementia diagnoses were based on DSM-5 criteria. Subjects were included if they had complete data on all biomarkers, age, and sex.

### 2.2 Measurement of CSF and blood biomarkers; APOE genotyping

CSF was collected by lumbar puncture using a 21-gauge needle in 10-ml polypropylene tubes. Part of the CSF was used for routine analysis including leukocyte count, erythrocyte count, glucose concentration, and total protein concentration. Within 2 h, the remaining CSF was centrifuged at 2000 *g* for 10 min at room temperature (RT) to eliminate cells, transferred to new polypropylene tubes, and stored at −80 °C until biomarker analysis. To obtain plasma, blood samples (5 ml) were collected from all patients after over-night fasting in EDTA K2-coated tubes and immediately centrifuged (3700 *g*, 20 min, RT). Plasma aliquots were frozen at −80 °C until blind assessments.

CSF Aβ40, Aβ42, tTau, and pTau181 were evaluated using commercially available kits using the Lumipulse® G600II platform (Fujirebio). Cut-off values employed for AD diagnosis were the following (normal values are reported): Aβ42 >599 pg/ml; tTau <404 pg/ml; pTau <56.5 pg/ml; Aβ42/40 ratio >0.069; Aβ42/tTau ratio >1.275; Aβ42/pTau ratio >8.1.

Plasma Aβ40, Aβ42, pTau181, pTau217 and NfL were measured by using commercially available kits with the Lumipulse® G600II platform (Fujirebio).

To analyze APOE genotype, total DNA was extracted from peripheral blood using a commercial DNA extraction kit (Qiagen, Venlo, Netherlands), and the genotype was determined using DiaPlexQ™ Apolipoprotein E (ApoE) Genotyping Kit (SolGent, Daejeon, Korea).

### 2.3 Statistical analysis

Summary statistics are presented as mean ± standard deviation. Subjects within the AD continuum were identified using the CSF Aβ42/40 ratio, with a cut-off value of 0.069. For comparing distributions between subjects in the AD continuum and non-AD subjects, either the *t*-test or Wilcoxon test were employed based on the normality of the variables, assessed using the Shapiro-Wilk test. Categorical variables were analyzed using chi-square tests. The percentage increase in the median across the two groups was calculated, and effect sizes were determined using Cohen's *d*. Differences were adjusted for age and sex using ANCOVA. Spearman's *ρ* or Pearson's r coefficients were used to assess correlations between biomarkers depending on normality.

To evaluate the performance of different biomarkers, logistic regression models were built with amyloid status as the dependent variable and each biomarker as the predictors. For each model, *p*-values and standardized coefficients were calculated. Receiver Operating Characteristic (ROC) analysis was conducted to determine the optimal cut-off values, based on Youden index maximization and on the two cut-offs approach proposed by Schindler et al. [11]. Additionally, optimal sensitivity, specificity, as well as positive and negative predictive values were computed. A robustness analysis was carried out simulating the performances of the different BBADs with increasing random coefficient of variations in their measurement. Comparisons of the Area Under the Curve (AUC) for blood biomarkers were performed using the DeLong test. Lastly, linear regression models were employed to evaluate predictors of the MMSE scores.

## 3 Results

The characteristics of the recruited sample are detailed in Table 1: *n* = 102 consecutive subjects were included, almost equally splitted according to gender. The mean age of the participants was 71.0 ± 7.6 years, with an average education level of 9.6 ± 3.9 years, and a mean MMSE of 23.0 ± 4.3. The majority of the participants were ApoE *ε*4 negative, whereas 39% (*n* = 40) were ApoE *ε*4 carriers. Nearly half of the sample (47%, *n* = 43) was diagnosed with MCI, and the remaining 53% (*n* = 49) had a diagnosis of dementia. Diagnostic information was not available for *n* = 10 patients.

**Table 1:** Baseline characteristics of the sample.

	All (*n* = 102)	A- (*n* = 33)	*A*+ (*n* = 69)	
Variable	Mean ± sd	Mean ± sd	Mean ± sd	p-value
Sex				ns
Male	50 (49%)	12 (36%)	38 (55%)	
Female	52 (51%)	21 (64%)	31 (45%)	
Education	9.6 ± 3.9	10.4 ± 3.1	9.4 ± 4.2	ns
Age	71.0 ± 7.6	66.9 ± 8.6	73.0 ± 6.1	<0.001
MMSE	23.0 ± 4.3	24.2 ± 4.16	22.4 ± 4.21	ns
ApoE ε4				< 0.001
negative	62 (61%)	27 (82%)	35 (51%)	
positive	40 (39%)	6 (18%)	34 (49%)	
Diagnosis				
MCI	43 (47%)	9 (36%)	34 (51%)	ns
Dementia	49 (53%)	16 (64%)	33 (49%)	

*Notes: A* = amyloid status; MCI = Mild cognitive impairment.

There were no significant differences in sex, education, MMSE scores, or baseline diagnoses between amyloid-positive, as defined by CSF Aβ42/40 ratio (*A*+, *n* = 69, 67%), and amyloid-negative (A-, *n* = 33, 33%) subjects. However, *A*+ subjects were significantly more likely to be ApoE ε4 carriers (*p* < 0.001) and were, on average, older than A- subjects (73.0 ± 6.1 years vs*.* 66.9 ± 8.6 years, *p* < 0.001).

Using a CSF pTau181 cut-off of 56.5 pg/ml, *n* = 31 subjects were categorized as A-T_1_-, *n* = 2 as A-T_1_+, *n* = 8 as *A* + T_1_-, and *n* = 61 as *A* + T_1_+. Due to the small size of the A-T_1_+ and *A* + T_1_- groups, only the A status was considered in the subsequent analyses.

Significant differences were observed in all CSF and plasma biomarkers between A- and *A*+ subjects, except for plasma Aβ40 and NfL. However, after adjusting for age and sex, the difference in plasma Aβ42/40 ratio was no longer significant. As expected, *A*+ subjects exhibited significantly lower levels of Aβ42 and significantly higher levels of plasma pTau (both pTau181 and pTau217), as shown in Table 2.

**Table 2:** Differences in blood and CSF biomarkers.

Variable	Total sample (*n* = 102)	A- (*n* = 33)	*A*+ (*n* = 69)	Percentage difference	Effect size [95% CI]	p value	adjusted p
*CSF*							
Aβ42	688.39 ± 351.11	966.09 ± 410.34	555.58 ± 220.33	−39.5%	−1.4 [−1.9; −0.9]	<0.001	<0.001
tTau	542.95 ± 292.01	335.48 ± 139.22	643.63 ± 294.15	93.5%	1.2 [0.8; 1.7]	<0.001	<0.001
p Tau	89.55 ± 62.39	39.26 ± 16.14	113.60 ± 62.01	187.3%	1.4 [1; 1.9]	<0.001	<0.001
Aβ40	11,777.6 ± 4538.02	10,352.06 ± 3782.77	12,459.38 ± 4732.43	17.4%	0.5 [0; 0.9]	0.015	0.029
Aβ42/40	0.06 ± 0.02	0.09 ± 0.01	0.05 ± 0.01	−53.7%	−4.1 [−4.8; −3.4]	<0.001	<0.001
Aβ42/tTau	1.75 ± 1.4	3.15 ± 1.32	1.08 ± 0.82	−69.4%	−2.1 [−2.6; −1.5]	<0.001	<0.001
Aβ42/pTau	12.73 ± 11.06	26.08 ± 8.61	6.34 ± 4.37	−81.0%	−3.3 [−3.9; −2.6]	<0.001	<0.001
*Plasma*							
Aβ42	24.48 ± 9.97	30.91 ± 7.51	21.41 ± 9.56	−29.0%	−1.1 [−1.5; −0.6]	<0.001	<0.001
Aβ40	316.87 ± 116.69	347.99 ± 83.24	301.99 ± 127.55	−6.8%	−0.4 [−0.8; 0]	0.116	0.059*
pTau181	2.76 ± 1.67	1.68 ± 0.64	3.28 ± 1.77	100.0%	1.1 [0.6; 1.5]	<0.001	<0.001
pTau217	0.61 ± 0.55	0.18 ± 0.11	0.83 ± 0.56	411.2%	1.4 [0.9; 1.9]	<0.001	<0.001
Aβ42/40	0.09 ± 0.09	0.09 ± 0.01	0.09 ± 0.11	−17.0%	0.0 [−0.4; 0.4]	<0.001	0.929*
NfL	38.97 ± 31.12	47.73 ± 34.24	34.78 ± 28.84	−14.1%	−0.4 [−0.8; 0]	0.096	0.048*

*Notes:* CSF = cerebrospinal fluid. NfL = Neurofilament light chain. All variables values are expressed in pg/ml. Adjusted p values have been obtained after adjusting for age and sex. * = not significant after adjustment for False Discovery Rate and Bonferroni.

Among blood biomarkers, pTau217 showed the highest percentage increase in median values (411.2%), followed by pTau181 (100.0%). Effect sizes for these differences were generally large.

**Fig. 1:**
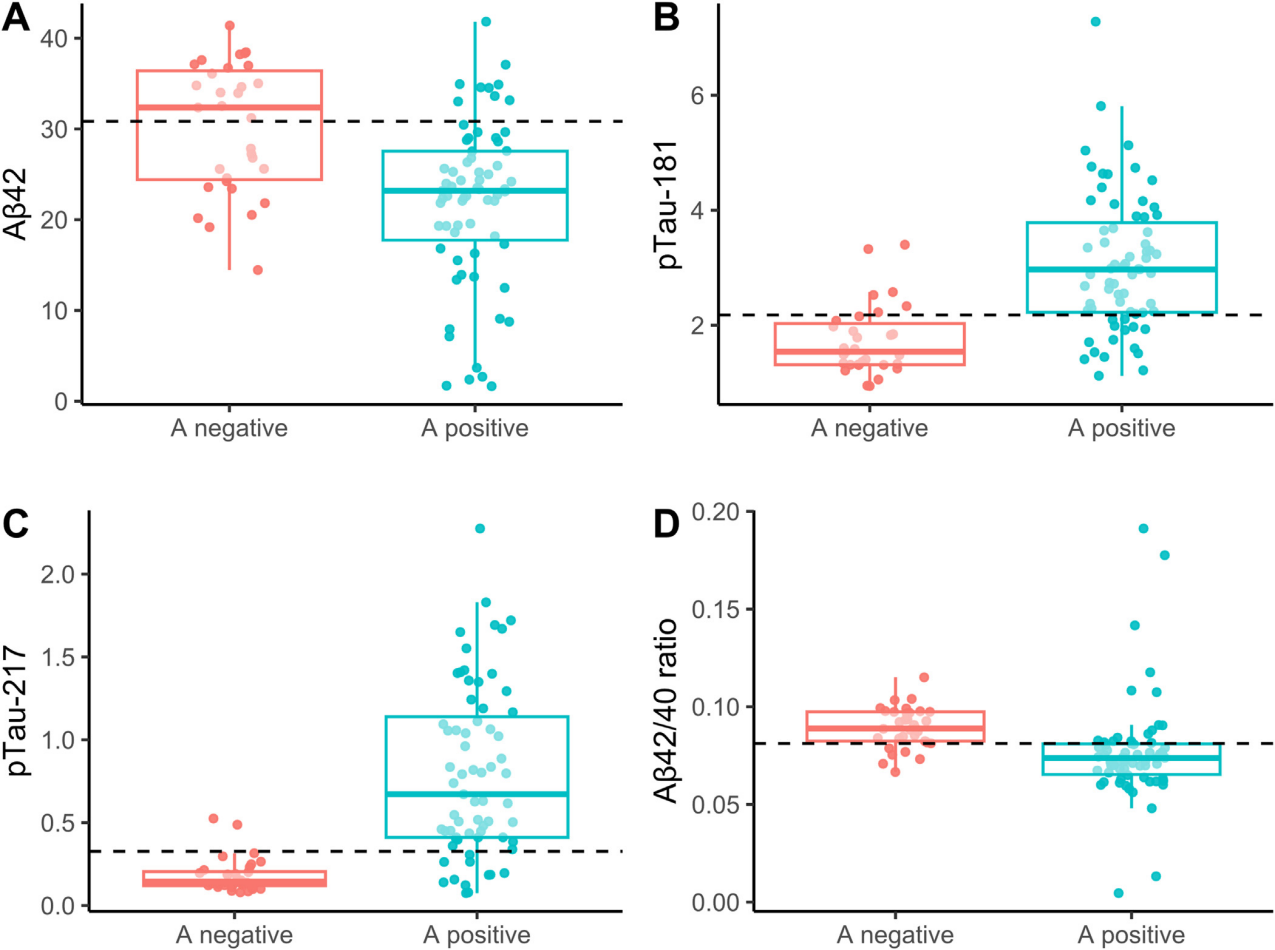
Boxplots for plasma Aβ42, pTau181, pTau217 and Aβ42/40 ratio, according to CSF amyloid status. Outliers (above 95th and under 5th percentiles) have been excluded. The horizontal line indicates the optimal cut-off based on maximization of the Youden index.

The results of the logistic models with A status as dependent variable and each biomarker as predictor are shown in Table 3. Notably, the performance of the plasma Aβ42/40 ratio was adversely affected by outliers. When we re-ran the model excluding outliers (outside of ± 3 standard deviations) the performance of Aβ42/40 improved significantly. Boxplots for plasma Aβ42, pTau181, pTau217, and the Aβ42/40 ratio are presented in Fig. 1, excluding outliers.

**Table 3:** Logistic model and diagnostic performances of CSF and blood biomarkers.

Variable	St. coefficient	Variable p	Cut-off	AUC [95% CI]	Optimal SE	Optimal SP	PPV	NPV
*CSF*								
Aβ42	−1.82	<0.001	759.001	0.844 [0.764, 0.923]	0.866	0.667	0.84	0.69
Aβ40	0.52	0.036	10,894.925	0.641 [0.525, 0.758]	0.597	0.697	0.80	0.46
tTau	2.27	<0.001	366.999	0.860 [0.784, 0.936]	0.896	0.727	0.87	0.77
pTau	4.61	<0.001	63.300	0.931 [0.879, 0.983]	0.851	0.939	0.97	0.76
Aβ42/tTau	−2.57	<0.001	1.843	0.935 [0.887, 0.983]	0.910	0.879	0.94	0.83
Aβ42/pTau	−3.88	<0.001	12.997	0.978 [0.954, 1.000]	0.940	0.939	0.97	0.89
*Plasma*								
Aβ42	−1.37	<0.001	30.836	0.781 [0.684, 0.877]	0.881	0.576	0.81	0.68
Aβ40	−0.44	0,072	195.978	0.602 [0.486, 0.717]	0.224	1.000	1.00	0.38
pTau181	3.16	<0.001	2.180	0.869 [0.796, 0.942]	0.791	0.818	0.90	0.64
pTau217	5.37	<0.001	0.327	0.911 [0.853, 0.969]	0.836	0.939	0.97	0.74
Aβ42/40	−0.01	0.947	0.081	0.802 [0.713, 0.892]	0.746	0.818	0.89	0.61
Aβ42/40*	−1.49	<0.001	0.086	0.834 [0.748; 0.920]	0.778	0.818	0.89	0.66
NfL	−0.42	0.065	44.840	0.609 [0.484, 0.734]	0.866	0.424	0.75	0.61

*Notes:* CSF = cerebrospinal fluid. NfL = Neurofilament light chain. * = excluding outliers*.* All variables values are expressed in pg/ml.

**Fig. 2:**
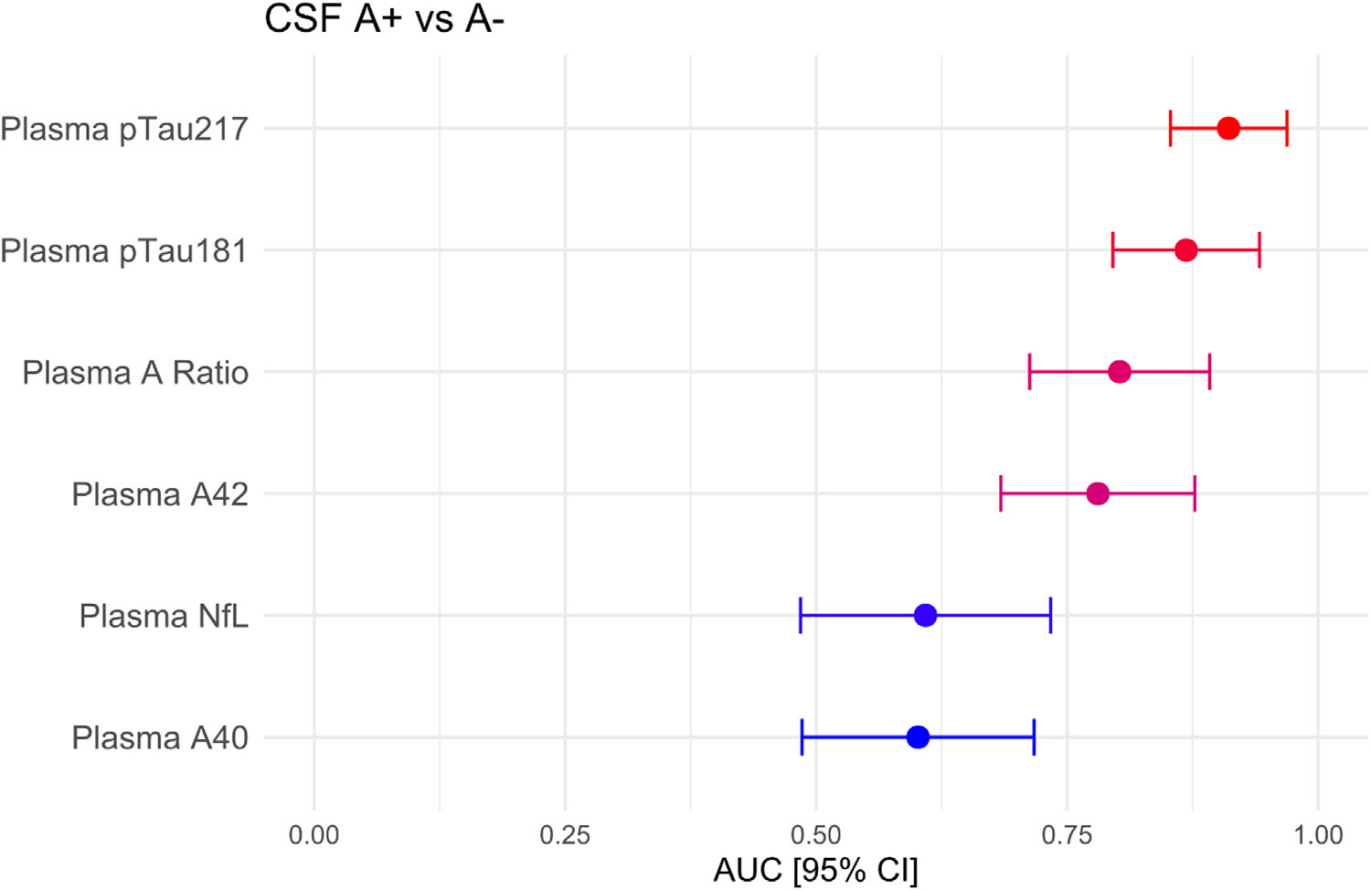
AUC with 95% confidence interval for the different plasma biomarkers in discriminating between amyloid positive and negative subjects.

Plasma biomarkers AUCs with their confidence intervals are displayed in Fig. 2. The results of the DeLong test for comparing these AUCs are shown in Table 4. pTau217 outperformed all other blood biomarkers except pTau181, although the difference with Aβ42 and Aβ42/40 ratio was not significant after FDR correction for multiple comparisons. Plasma pTau181 was only superior to plasma Aβ40 and NfL. A robustness analysis showed that the diagnostic performance of both pTau217 and pTau181 was maintained across a wide range of preclinical variability [adding up to a 0.25 random coefficient of variation (CV)]. Plasma pTau181 performance declined slightly more with increasing CV, while the performance of the plasma Aβ42/40 ratio quickly dropped (see **Figures S1** and **S2** in the supplementary material).

**Table 4:** p values of the DeLong tests to compare the different AUCs of plasma biomarkers.

	Aβ42	Aβ40	Aβ42/40	pTau-181	pTau-217	NfL
Aβ42	–	<0.001	0.677	0.205	0.028*	0.031*
Aβ40	<0.001	–	0.010	0.001	<0.001	0.946
Aβ42/40	0.677	0.010	–	0.299	0.045*	0.012
pTau-181	0.205	0.001	0.299	–	0.174	0.001
pTau-217	0.028*	<0.001	0.045*	0.174	–	<0.001
NfL	0.031*	0.946	0.012	0.001	<0.001	–

*Notes:* NfL = Neurofilament light chain. * = not significant after FDR correction.

A sensitivity analysis that included only MCI subjects resulted in similar AUCs, with lower NPV, possibly influenced by the higher prevalence of AD in this subgroup (79%). These analyses are provided in **Table S1** in the supplementary material. The results of sensitivity analyses using CSF Aβ42/pTau ratio are presented in Table S2-S4 in the supplementary material. These yielded similar results, with the exception of the loss of significance of plasma NfL, and the considerably lower performance of plasma Aβ42/40 ratio. Notably, the performance of pTau217 was similar, with an AUC of 0.911, with an excellent NPV of 0.91, while retaining a good PPV of 0.82.

**Fig. 3:**
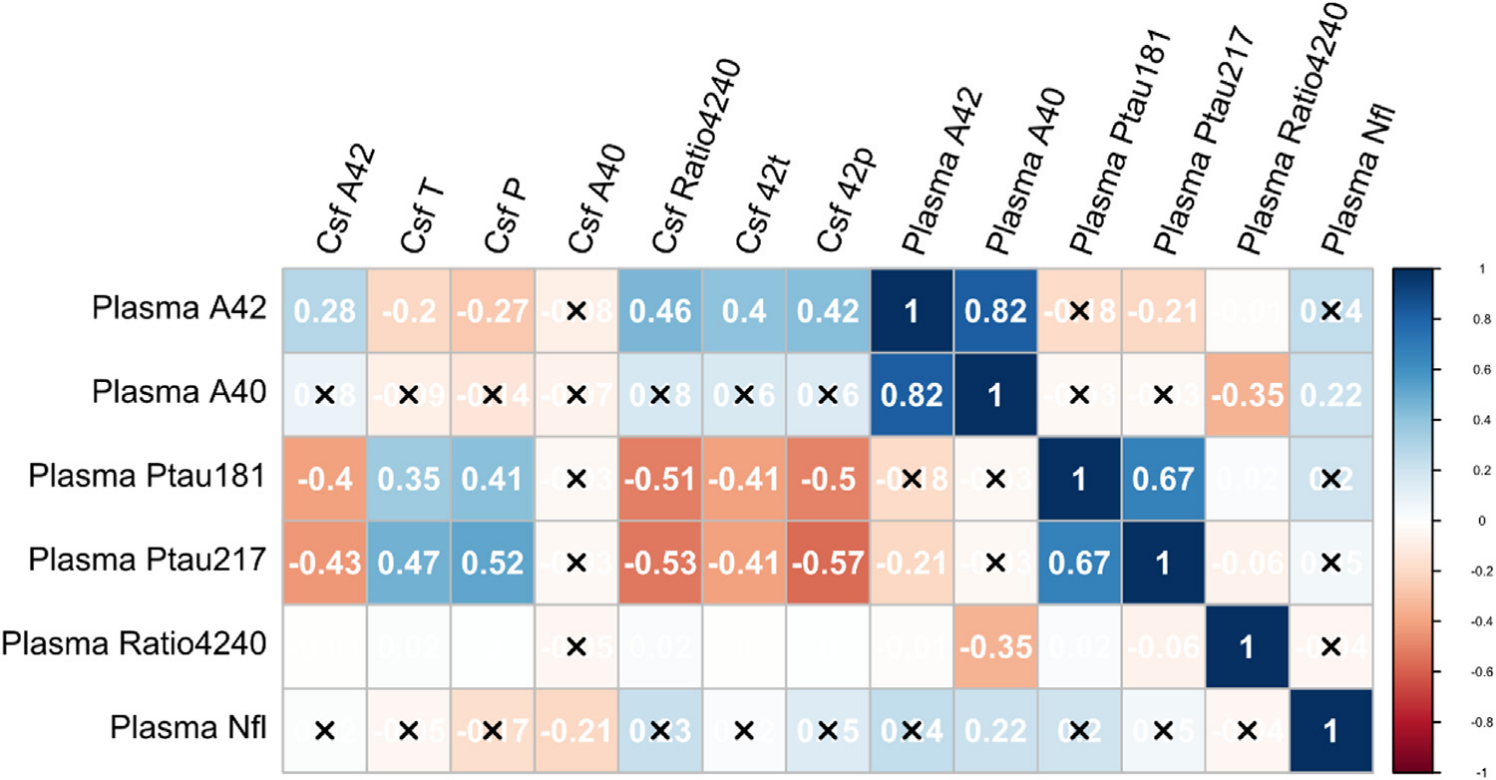
Spearman correlation coefficients between plasma biomarkers (rows) and all plasma and CSF biomarkers (columns). Non-significant correlations have been suppressed with an X.

Correlations between plasma biomarkers and all other biomarkers are illustrated in Fig. 3**.**

**Fig. 4:**
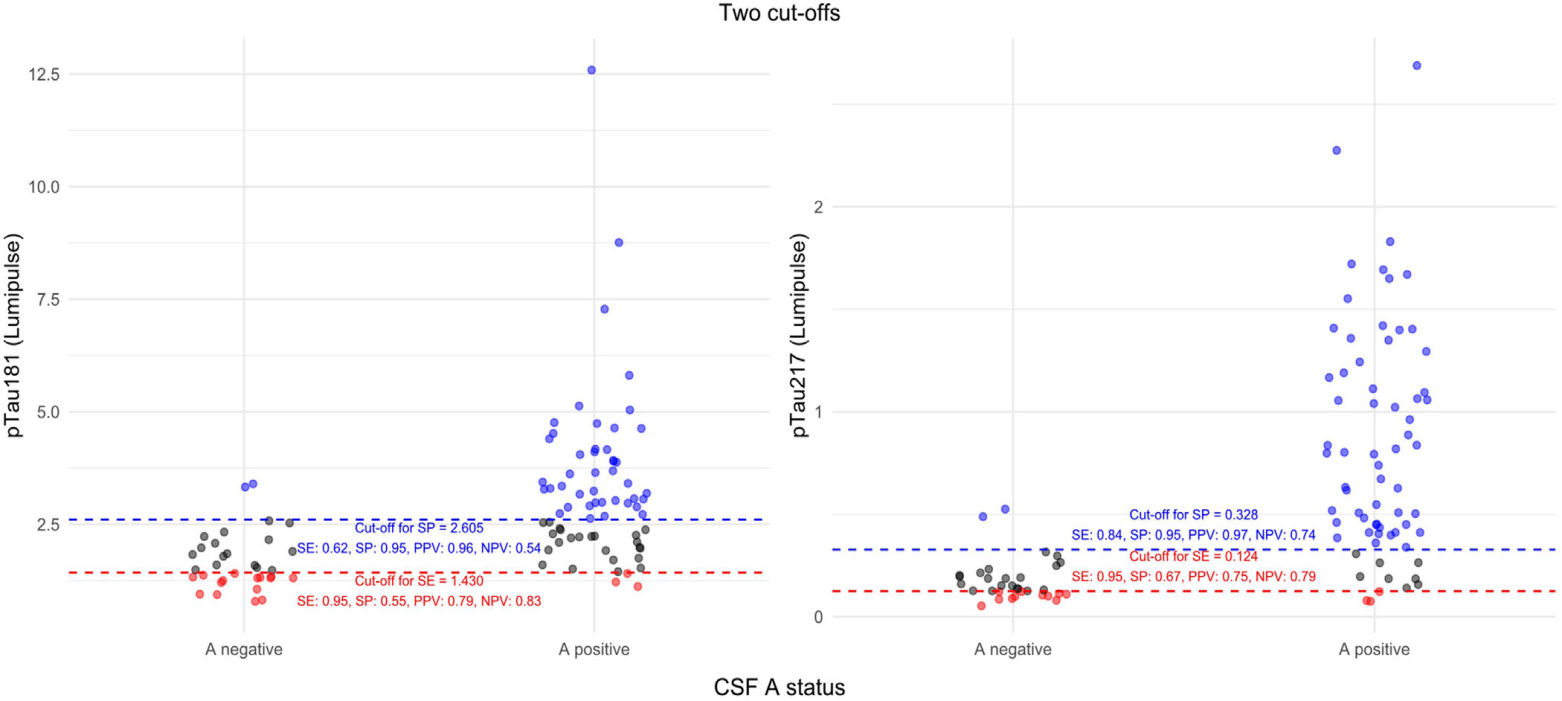
Results of a two cut-offs approach for plasma pTau181 and pTau217.

The results of a two cut-offs approach for plasma pTau181 and pTau217 are presented in Fig. 4**.** For both BBADs, the 95% specificity cut-off (2.60 pg/nL and 0.328 pg/nL respectively) yielded excellent PPVs (0.96 and 0.97). Conversely, the 95% sensitivity cut-off showed only limited NPVs (0.83 and 0.79).

We performed the same analysis with CSF Aβ42/pTau ratio, showing similar PPVs for the high-specificity cut-offs, and higher NPVs for the high-sensitivity cut-offs (respectively 0.87 for pTau181 and 0.92 for pTau217). These results are shown in **Figure S3.**

Furthermore, we conducted linear regression models with baseline MMSE as the dependent variable. Each model included one plasma biomarker as a predictor, along with age, sex, ApoE status, diagnosis, and the interaction between the plasma biomarker and diagnosis. The findings are summarized in Table 5. Although all models were significant, among plasma biomarkers only pTau181 and pTau217 emerged as significant predictors of baseline MMSE, both with negative coefficients, as expected. A diagnosis of MCI was a significant and consistent predictor of higher MMSE scores. By contrast, age, ApoE, sex, and the interactions between each variable and diagnosis were not significant predictors. When including education among the predictors, the significance of the results did not change (see **Table S5** in the supplementary material). Education was a marginally significant predictor only in the model with plasma pTau217.

**Table 5:** p values and unstandardized coefficients of different linear models to predict MMSE.

		Variable		Initial group [MCI]		Age		ApoE4 [positive]		Sex [female]		Variable*Initial group [MCI]	
BBAD	Model p	Coefficient [SE]	p	Coefficient [SE]	p	Coefficient [SE]	p	Coefficient [SE]	p	Coefficient [SE]	p	Coefficient [SE]	p
Aβ42	<0.001	0.067 [0.055]	0.233	5.333 [2.127]	0.014	−0.115 [0.058]	0.051	−0.256 [0.829]	0.758	−1.491 [0.809]	0.069	−0.05 [0.082]	0.542
Aβ40	<0.001	0.001 [0.005]	0.865	5.511 [2.306]	0.019	−0.117 [0.06]	0.055	−0.107 [0.847]	0.900	−1.386 [0.812]	0.092	−0.004 [0.007]	0.558
pTau-181	<0.001	−0.966 [0.364]	0.010	2.909 [1.506]	0.057	−0.079 [0.056]	0.163	0.235 [0.802]	0.770	−1.436 [0.763]	0.063	0.432 [0.455]	0.345
pTau-217	<0.001	−2.529 [0.942]	0.009	3.389 [1.217]	0.007	−0.073 [0.057]	0.205	0.265 [0.805]	0.743	−1.2 [0.774]	0.125	0.675 [1.37]	0.624
Aβ42/40	<0.001	3.344 [4.53]	0.463	1.47 [2.6]	0.573	−0.108 [0.06]	0.074	0.17 [0.856]	0.843	−1.574 [0.803]	0.053	33.563 [29.915]	0.265
NfL	<0.001	0.005 [0.017]	0.755	4.921 [1.427]	0.001	−0.114 [0.06]	0.063	−0.137 [0.834]	0.870	−1.484 [0.82]	0.074	−0.02 [0.034]	0.556

## 4 Discussion

BBAD come of age in a moment in which disease-modifying drugs are getting increasingly real. We are in strong need of robust and non-invasive biomarkers of state, when thinking, not only to candidate patients to novel therapies, but also to follow them over time. Over the years, the assessment methodology of core biomarkers of AD became more robust and we are now concretely facing the ethical dilemma of easily testing since early prodromal stages of the amyloid as a disease, stratifying the actual risk of developing in the future major cognitive complains and functional decline. This approach will lead to implement a plethora of promising multimodal preventive strategies that may eventually prove to be less costly with respect to anti-amyloid antibodies [12].

Plasma pTau217 and plasma pTau181, as measured with Lumipulse®, showed good and robust predictors of CSF amyloid status in a real-world setting of our memory clinic. Plasma Aβ42/40 ratio, on the contrary, had slightly lower performances but may lack somehow of robustness.

The prevalence of amyloid pathology in our cohort was 67%, in line with what could be expected in a memory clinic. In this setting, a cut-off of 0.32 pg/ml for pTau217 and 2.18 pg/ml for pTau181 show a high PPV (0.97 and 0.90 respectively), but a suboptimal NPV (0.74 and 0.64 respectively). With a SP of 0.93 and a SE of 0.83, the performance of pTau217 is quite close to a clinically acceptable performance for a confirmatory test (90% SP and SE), as suggested in a recent consensus on plasma biomarkers [11]. Indeed, in our secondary care setting a positive pTau217 test could be acceptable for confirmation of amyloid pathology in most patients, bearing in mind that a negative test does not definitively rule out amyloid pathology owing to the low NPV, requiring additional CSF test or amyloid PET, or, perhaps, a second BBAD [8]. Moreover, plasma pTau217 had the highest fold of change (more than 4 times) in *A*+ subjects, and the highest module of the standardized coefficient when used as a cross-sectional predictor of MMSE scores.

The same recent consensus suggested a two cut-offs approach for BBADs, with a high-specificity cut-off (95% specificity) to rule in amyloid pathology and a high-sensitivity cut-off (95% sensitivity) to rule it out [11]. We calculated such cut-offs for the most promising BBAD, i.e. pTau181 and pTau217. For plasma pTau181, these are 2.6 and 1.4 pg/ml respectively, while for plasma pTau217 the cut-offs are 0.328 and 0.124 pg/ml. In both cases, the high-specificity cut-off is quite close to the optimal cut-off based on the maximization of the Youden index, possibly due to the high prevalence of amyloid pathology in our cohort. Moreover, while PPVs for the high-specificity cut-offs are excellent, NPVs for the high-sensitivity cut-offs are sub-optimal. Therefore, based on our results, one can imagine that the preferred use of pTau BBADs would be ruling in amyloid pathology.

With a slightly higher cut-off of 0.350 pg/mL pTau217 has a sufficient NPV to rule out a more advanced pathology along the AD continuum, as defined by the CSF Aβ42/pTau ratio. This is due to the expected lower prevalence of AD subjects identified by using this ratio (around 50%) compared to the prevalence of *A*+ subjects.

Our results are in line with the most recent literature on Lumipulse® biomarkers. A study on patients from a memory clinic, also including cognitively unimpaired individuals, showed that plasma p-Tau 217 outperformed other BBAD, with an AUC of 0.94 [0.92–0.97], with a cut-off slightly lower than ours, of 0.25 pg/ml [13]. Plasma p-Tau 217 as measured with Lumipulse® has demonstrated a very high AUC of 0.95 [0.93–0.98] in distinguishing AD from other neurodegenerative diseases, with a slightly lower cut-off of 0.27 pg/ml [14]. In the same paper, the correlation of plasma pTau217 with CSF pTau was practically the same as in our study. In another recent study Lumipulse® plasma pTau217 showed a higher AUC of 0.94–0.97 in distinguishing *A*+ from A- subjects, with a lower cut-off of 0.23 pg/ml [15]; this result was similar to a study using amyloid PET as standard of truth [16]. The latter also reported the results of a two cut-offs approach, which are quite similar to the ones we identified (0.185 and 0.324 pg/ml respectively) [16].

Studies on plasma pTau181 with Lumipulse® have given mixed results. For instance, in the study by Wilson the AUC for this biomarker was 0.96 [0.91–0.99], with an optimal cut-off of 2.35 pg/ml, not far from the one we found [17]. However, the authors used a slightly higher cut-off for CSF Aβ42/40 ratio of 0.091, and more than half of their sample consisted of cognitively unimpaired individuals, which may partially explain their better diagnostic performances. Another recent study, with a higher proportion of AD subjects, showed an AUC of 0.89 [0.81–0.96], more similar to our findings [18]. Also the recent study by Arranz in a memory clinic setting was similar to ours, with a AUC of 0.88 [0.84–0.92], with an optimal cut-off of 2.12 pg/ml, very close to ours [13]. Another work on a large combined cohort from two memory clinics showed similar results (AUC 0.86 [0.82–0.90], cut-off 2.08 pg/ml) [19]. On the contrary, in a recent study by Janelidze the performance of plasma pTau181 in MCI subjects was much lower (AUC 0.69 [0.60–0.78]), and inferior to that of pTau217 [20]. When we restricted the analysis to MCI subjects, the performance of plasma pTau181 actually improved (AUC 0.88 [0.77–0.99]), although with a broader confidence interval due to the smaller sample size. In another study published this year, the performance of Lumipulse® plasma pTau181 was similar to ours (AUC 0.81 [0.73–0.89]), with a lower cut-off of 2.07 pg/ml. The study included a higher percentage of MCI subjects, and less AD patients compared to our work [21]. In a cohort comprising mostly cognitively unimpaired individuals, the AUC of Lumipulse® plasma pTau181 in predicting positive amyloid-PET was not great, with an AUC of 0.74 [0.67–0.80], with an optimal cut-off of 2.46 pg/ml [22]. Finally, a study on cognitively unimpaired subjects confirmed a suboptimal performance of plasma pTau181, with an AUC of 0.73 [0.66–0.80].

Not many works exist on the performance of Lumipulse® plasma Aβ42/40 ratio. The paper by Arranz, on consecutive subjects in a memory clinic, showed a good AUC of 0.88 [0.84–0.92], with an optimal cut-off of 0.078 [13]. This was in line with the recent study by Bellomo, showing an AUC of 0.86 [0.83–0.90], with a cut-off of 0.080 [19]. A study by Figdore, including mostly cognitively unimpaired individuals from the Mayo Clinic Study of Aging, shows an AUC of 0.81 [0.75–0.86] for the prediction of positive amyloid-PET, with a cut-off of 0.077, similar to what we found. In the same study, the performance of the SIMOA plasma Aβ42/40 was greatly inferior to the Lumipulse® assay [22]. In another work on cognitively unimpaired volunteers, the plasma Aβ42/40 ratio was even superior to plasma pTau181, with an AUC of 0.89 [0.86–0.94] [23]. However, the authors did not perform a robustness analysis, which in our work seems to undermine the actual usefulness of such a BBAD.

It is worth noticing that plasma Aβ42/40 ratio may reach a plateau early in AD, as shown by a recent work [24]. However, another independent group found that plasma Aβ42/40 ratio keeps declining even later in the disease [25]. Due to the cross-sectional nature of our study, we could not ascertain whether plasma Aβ42/40 ratio would be useful to differentiate different stages of the disease.

We should remember that certain pre-analytical factors are known to influence BBAD values. For instance, plasma pTau181 and pTau217, as well as Aβ42 and Aβ40 correlate with kidney function, being higher with decreasing renal function. However, the Aβ42/40 ratio may be less influenced by filtration rate [13,19,26]. Creatinine and BMI seem to have a significant, albeit poorly relevant effect on NfL measurement [27]. It is plausible that other factors may add some degree of variation in BBAD concentration, such as centrifugation [28]. Our robustness analysis shows that the performances of both plasma pTau are quite constant for up to 25% of added random variation, while the performance of plasma Aβ42/40 lacks such robustness. For reference, the study by Bellomo and colleagues showed that intra- and inter-assay CV for plasma pTau181, Aβ42 and Aβ40 measured with Lumipulse® are all below 10% [19].

Taken all these results together, it could be argued that plasma pTau217 could represent the single BBAD of choice in a memory clinic setting, with a slightly better performance than plasma pTau181. Aβ42/40 ratio performance was not only slightly worse but, more importantly, limited by its lack of robustness. Finally, NfL performance in detecting *A*+ subjects in a real-world setting is quite poor, in keeping with its known poor specificity [29].

Our study has some limitations. Its real-world setting implies that clinical data have not been systematically collected in a standardized way. We analyzed the data of consecutive patients undergoing lumbar puncture for clinical indication at our memory clinic, which means that our sample is clinically heterogeneous; among non-AD patients, it is possible that some may have been not neurodegenerative at all. Nevertheless, our results are mostly aligned with the literature, especially with other real-world studies. Due to the low size of the subgroups, we could not perform analyses across the whole AT continuum, but only evaluate subjects based on their A status

However, a sensitivity analysis using the CSF Aβ42/pTau ratio did not significantly change our results.

Finally, we acknowledge that the optimal cut-offs we found need further validation, due to our limited sample size.

## 5 Conclusion

Plasma pTau217, as measured with Lumipulse®, may be considered as the leading candidate for a core BBAD to confirm *A*+ in a memory clinic setting, offering a practical and less invasive alternative to traditional CSF. Plasma pTau181 may be a less ideal alternative, while Aβ42/40, albeit promising, seems to lack necessary robustness. Future research should focus on refining these BBAD, exploring their utility across various stages of cognitive impairment, and establishing standardized guidelines for their clinical application.

## Declaration of generative AI and AI-assisted technologies in the writing process

None used.

## Ethical standards

The study protocol was approved by the ethical committee Comitato Etico Monza e Brianza, Italy; protocol CAPE (ID: 3974) and protocol AGAINST-AD (ID: 1881). The study has been performed in accordance with the ethical standards laid down in the 1964 Declaration of Helsinki and its later amendments.

## Funding

AGAINST-AD (PRIN 2017; Prot. 2017PFYK27).

## Data availability statement

The data of this manuscript are available upon request to the corresponding author.

## Author contributions

Conceptualization and methodology: FEP, EC, LT, CPZ. Formal analysis and investigation: FEP, EC, CPZ, GR, NO, SA. Writing – original draft preparation: FEP, EC, LT. Writing – review and editing: all authors.

## Declaration of competing interest

On behalf of all authors, the corresponding author states that there is no conflict of interest regarding the publication of this article in Journal of Prevention of Alzheimer's Disease.

## Appendix Supplementary materials

Image, application 1
