# Supplementary material for: Core blood biomarkers of Alzheimer's disease: A single-center real-world performance study
Source: J Prev Alzheimers Dis. 2025 Jan 1;12(2):100027. doi: 10.1016/j.tjpad.2024.100027 (PMC12183927; doi:10.1016/j.tjpad.2024.100027)
Supplement: Supplementary file 1 [file mmc1.docx]

**Figure S1.** AUC variation of the different plasma biomarkers with increasing random coefficient of variation.

**
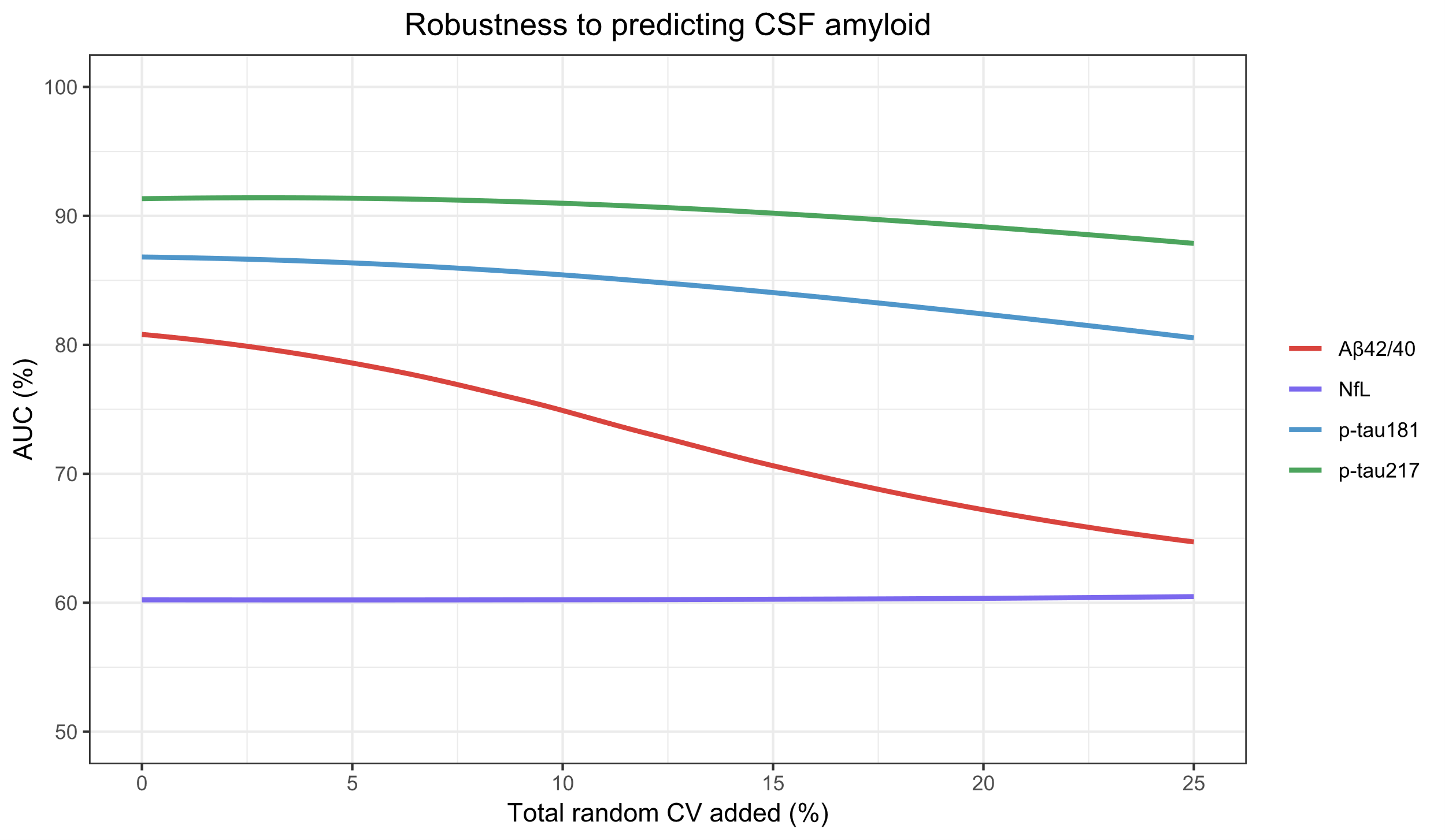
**

**Figure S2**. AUC variation with confidence intervals of the different plasma biomarkers with increasing random coefficient of variation.


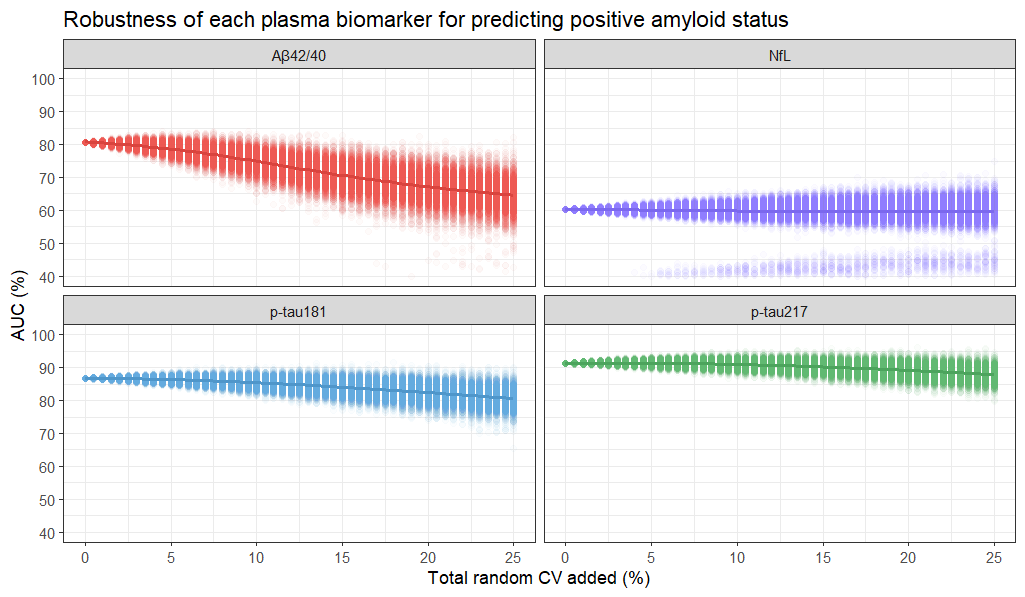


**Figure S3.** Results of a two cut-offs approach for plasma pTau181 and pTau217 with CSF Aβ42/p-Tau to define AD.

**
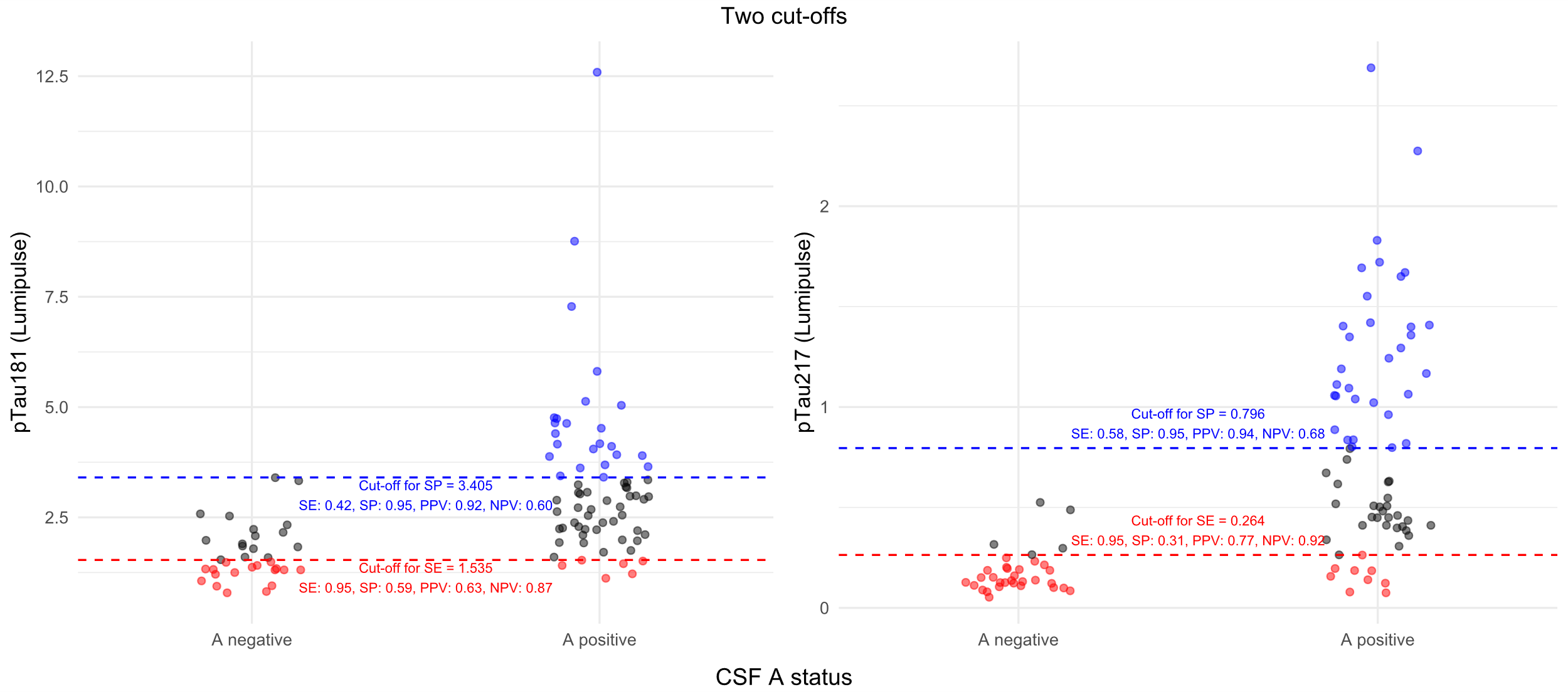
**

**Table S1.** Logistic model and diagnostic performances of CSF and blood biomarkers in MCI subjects using CSF Aβ42/40 ratio to define amyloid pathology.

| Variable | St. coefficient | Variable p | Cut-off | AUC [95% CI] | Optimal SE | Optimal SP | PPV | NPV |
| --- | --- | --- | --- | --- | --- | --- | --- | --- |
| *CSF* |  |  |  |  |  |  |  |  |
| Aβ42 | -1.24 | 0.021 | 907.312 | 0.739 [0.531, 0.946] | 0.912 | 0.556 | 0.88 | 0.63 |
| Aβ40 | 0.47 | 0.271 | 7941.923 | 0.614 [0.392, 0.837] | 0.882 | 0.444 | 0.85 | 0.50 |
| tTau | 2.33 | 0.012 | 430.907 | 0.899 [0.803, 0.994] | 0.735 | 1.000 | 1.00 | 0.50 |
| pTau | 3.64 | 0.005 | 63.793 | 0.925 [0.844, 1.000] | 0.882 | 1.000 | 1.00 | 0.69 |
| Aβ42/tTau | -1.68 | 0.007 | 1.963 | 0.938 [0.862, 1.000] | 0.912 | 0.889 | 0.97 | 0.73 |
| Aβ42/pTau | -3.63 | 0.004 | 1.722 | 0.971 [0.916, 1.000] | 0.971 | 0.889 | 0.97 | 0.89 |
| *Plasma* |  |  |  |  |  |  |  |  |
| Aβ42 | -1.37 | 0.025 | 31.061 | 0.745 [0.566, 0.924] | 0.824 | 0.667 | 0.90 | 0.55 |
| Aβ40 | -0.53 | 0.254 | 330.847 | 0.565 [0.382, 0.749] | 0.441 | 0.889 | 0.94 | 0.31 |
| pTau181 | 4.49 | 0.008 | 2.095 | 0.882 [0.773, 0.992] | 0.794 | 0.889 | 0.96 | 0.57 |
| pTau217 | 6.00 | 0.013 | 0.327 | 0.899 [0.806, 0.992] | 0.788 | 1.000 | 1.00 | 0.56 |
| Aβ42/40 | -0.35 | 0.311 | 0.084 | 0.746 [0.562, 0.930] | 0.818 | 0.667 | 0.90 | 0.50 |
| NfL | -0.24 | 0.484 | 20.175 | 0.579 [0.359, 0.799] | 0.364 | 0.889 | 0.92 | 0.28 |

**Table S2.** Baseline characteristics of the sample according to AT profile.

|  | All (n=102) | AT- (n=49) | AT+ (n=53) |  |
| --- | --- | --- | --- | --- |
| **Variable** | **Mean ± sd** | **Mean ± sd** | **Mean ± sd** | **p-value** |
| Sex |  |  |  | ns |
| ... Male | 50 (49%) | 23 (47%) | 27 (51%) |  |
| ... Female | 52 (51%) | 26 (53%) | 26 (49%) |  |
| Education | 9.6 ± 3.9 | 9.3 ± 4.2 | 9.8 ± 3.8 | ns |
| Age | 71.0 ± 7.6 | 70.0 ± 8.9 | 72.0 ± 6.0 | ns |
| MMSE | 23.0 ± 4.3 | 24.0 ± 3.8 | 22.0 ± 4.5 | ns |
| ApoE ε4 |  |  |  | < 0.001 |
| ... negative | 62 (61%) | 39 (80%) | 23 (43%) |  |
| ... positive | 40 (39%) | 10 (20%) | 30 (57%) |  |
| Diagnosis |  |  |  |  |
| ... MCI | 43 (42%) | 15 (38%) | 28 (53%) | ns |
| ... Dementia | 49 (53%) | 24 (62%) | 25 (47%) |  |

*Notes:* A = amyloid status; MCI = Mild cognitive impairment.

**Table S3.** Differences in blood and CSF biomarkers according to AT profile.

| **Variable** | **Total sample (n=102)** | **AT- (n=49)** | **AT+ (n=53)** | **Percentage difference** | **Effect size [95% CI]** | **p value** | **adjusted p** |
| --- | --- | --- | --- | --- | --- | --- | --- |
| *CSF* |  |  |  |  |  |  |  |
| Aβ42 | 688.39 ± 351.11 | 884.10 ± 387.96 | 507.45 ± 176.21 | -38.8% | -1.3 [-1.7; -0.8] | <0.001 | <0.001 |
| tTau | 542.95 ± 292.01 | 356.63 ± 151.03 | 718.52 ± 284.51 | 113.7% | 1.6 [1.1; 2] | <0.001 | <0.001 |
| pTau | 89.55 ± 62.39 | 45.07 ± 19.44 | 130.67 ± 60.21 | 173.5% | 1.9 [1.4; 2.4] | <0.001 | <0.001 |
| Aβ40 | 11777.6 ± 4538.02 | 11113.08 ± 4292.97 | 12391.96 ± 4710.25 | 7.8% | 0.3 [-0.1; 0.7] | 0.137 | 0.160 |
| Aβ42/40 | 0.06 ± 0.02 | 0.08 ± 0.02 | 0.04 ± 0.01 | -49.4% | -2.4 [-3; -1.9] | <0.001 | <0.001 |
| Aβ42/tTau | 1.75 ± 1.4 | 2.80 ± 1.36 | 0.77 ± 0.31 | -71.6% | -2.1 [-2.6; -1.6] | <0.001 | <0.001 |
| Aβa42/pTau | 12.73 ± 11.06 | 21.80 ± 9.62 | 4.34 ± 1.73 | -81.1% | -2.6 [-3.1; -2] | <0.001 | <0.001 |
| *Plasma* |  |  |  |  |  |  |  |
| Aβ42 | 24.48 ± 9.97 | 28.05 ± 10.15 | 21.18 ± 8.65 | -18.40% | -0.7 [-1.1; -0.3] | <0.001 | <0.001 |
| Aβ40 | 316.87 ± 116.69 | 335.01 ± 111.18 | 300.10 ± 120.17 | -6.30% | -0.3 [-0.7; 0.1] | 0.174 | 0.130 |
| pTau181 | 2.76 ± 1.67 | 1.93 ± 0.86 | 3.53 ± 1.87 | 78.20% | 1.1 [0.7; 1.5] | <0.001 | <0.001 |
| pTau217 | 0.61 ± 0.55 | 0.28 ± 0.39 | 0.93 ± 0.50 | 438.70% | 1.4 [1; 1.9] | <0.001 | <0.001 |
| Aβ42/40 | 0.09 ± 0.09 | 0.09 ± 0.02 | 0.09 ± 0.12 | -14.30% | 0.1 [-0.3; 0.5] | <0.001 | 0.758 |
| NfL | 38.97 ± 31.12 | 45.90 ± 39.29 | 32.56 ± 19.21 | -3.10% | -0.4 [-0.8; 0] | 0.227 | 0.030 |

*Notes:* CSF = cerebrospinal fluid. NfL = Neurofilament light chain. All variables values are expressed in pg/ml. Adjusted p values have been obtained after adjusting for age and sex.

**Table S4.** Logistic model and diagnostic performances of CSF and blood biomarkers in predicting AT profile.

| Variable | St. coefficient | Variable p | Cut-off | AUC [95% CI] | Optimal SE | Optimal SP | PPV | NPV |
| --- | --- | --- | --- | --- | --- | --- | --- | --- |
| *CSF* |  |  |  |  |  |  |  |  |
| Aβ42 | -2.25 | <0.001 | 606.500 | 0.844 [0.766. 0.923] | 0.77 | 0.84 | 0.83 | 0.77 |
| Aβ40 | 0.25 | 0.233 | 10895.000 | 0.573 [0.459. 0.686] | 0.59 | 0.59 | 0.60 | 0.59 |
| tTau | 2.68 | <0.001 | 435.005 | 0.899 [0.841. 0.957] | 0.88 | 0.80 | 0.82 | 0.88 |
| pTau | 6.16 | <0.001 | 72.999 | 0.970 [0.944. 0.997] | 0.92 | 0.92 | 0.92 | 0.92 |
| Aβ42/Aβ40 | -4.33 | <0.001 | 0.055 | 0.955 [0.921. 0.990] | 0.88 | 0.90 | 0.90 | 0.88 |
| Aβ42/tTau | -9.50 | <0.001 | 1.213 | 0.982 [0.964. 1.000] | 0.94 | 0.92 | 0.92 | 0.94 |
| *Plasma* |  |  |  |  |  |  |  |  |
| Aβ42 | -0.83 | 0.001 | 30.834 | 0.711 [0.608. 0.814] | 0.94 | 0.49 | 0.66 | 0.89 |
| Aβ40 | -0.33 | 0.118 | 386.568 | 0.584 [0.471. 0.697] | 0.82 | 0.37 | 0.58 | 0.67 |
| pTau181 | 2.34 | <0.001 | 2.535 | 0.849 [0.773. 0.924] | 0.77 | 0.84 | 0.83 | 0.77 |
| pTau217 | 2.28 | <0.001 | 0.350 | 0.911 [0.853. 0.970] | 0.92 | 0.80 | 0.82 | 0.91 |
| Aβ42/40 | -0.83 | 0.743 | 0.083 | 0.710 [0.604. 0.815] | 0.84 | 0.57 | 0.22 | 0.33 |
| Aβ42/40* | -0.38 | 0.167 | 0.035 | 0.707 [0.598; 0.815] | 0.85 | 0.56 | 0.66 | 0.79 |
| NfL | -0.55 | 0.040 | 45.130 | 0.578 [0.463, 0.694] | 0.94 | 0.39 | 0.62 | 0.86 |

*Notes:* CSF = cerebrospinal fluid. NfL = Neurofilament light chain. * = excluding outliers*.* All variables values are expressed in pg/ml.

**Table S5**. p values and unstandardized coefficients of different linear models to predict MMSE.

|  |  | Variable | | Initial group [MCI] | | Age | | ApoE4 [positive] | | Sex [female] | | Education | | Variable*Initial group [MCI] | |
| --- | --- | --- | --- | --- | --- | --- | --- | --- | --- | --- | --- | --- | --- | --- | --- |
| Variable | Model p | Coefficient [SE] | p | Coefficient [SE] | p | Coefficient [SE] | p | Coefficient [SE] | p | Coefficient [SE] | p | Coefficient [SE] | p | Coefficient [SE] | p |
| Plasma Aβ42 | <0.001 | 0.094 [0.06] | 0.119 | 6.164 [2.2] | 0.007 | -0.08[0.063] | 0.208 | -0.266 [0.851] | 0.755 | -1.131 [0.855] | 0.19 | 0.207 [0.112] | 0.069 | -0.09 [0.085] | 0.293 |
| Plasma Aβ40 | <0.001 | 0.002 [0.005] | 0.721 | 5.852 [2.353] | 0.015 | -0.078[0.066] | 0.237 | -0.071 [0.875] | 0.936 | -1.028 [0.859] | 0.235 | 0.223 [0.113] | 0.053 | -0.006 [0.007] | 0.422 |
| Plasma pTau181 | <0.001 | -1.012 [0.388] | 0.011 | 2.461 [1.635] | 0.137 | -0.042[0.061] | 0.495 | 0.139 [0.825] | 0.867 | -1.14 [0.809] | 0.163 | 0.207 [0.109] | 0.061 | 0.547 [0.483] | 0.261 |
| Plasma pTau217 | <0.001 | -2.659 [1.071] | 0.015 | 2.925 [1.383] | 0.038 | -0.037[0.063] | 0.554 | 0.153 [0.831] | 0.854 | -1.03 [0.819] | 0.213 | 0.228 [0.112] | 0.047 | 1.199 [1.526] | 0.435 |
| Plasma Aβ42/40 | <0.001 | 3.327 [4.531] | 0.465 | 1.809 [2.614] | 0.491 | -0.071[0.065] | 0.282 | 0.185 [0.891] | 0.836 | -1.266 [0.853] | 0.142 | 0.202 [0.113] | 0.079 | 28.27 [30.223] | 0.353 |
| Plasma Nfl | <0.001 | 0.007 [0.017] | 0.696 | 4.906 [1.464] | 0.001 | -0.073[0.066] | 0.272 | -0.121 [0.861] | 0.889 | -1.192 [0.874] | 0.177 | 0.224 [0.113] | 0.051 | -0.024 [0.034] | 0.487 |
